# Experiences of medication burden among patients with multimorbidity in rural China: a qualitative study

**DOI:** 10.3389/fmed.2026.1772444

**Published:** 2026-04-21

**Authors:** Yiting Lu, Fen Hu, Ming Yan, Mi Yao, Yu Xia, Junhai Zhen, Lingyan Wu, Yuling Tong, Yi Guo, Zhijie Xu

**Affiliations:** 1 Department of General Practice, Tongji University School of Medicine, Shanghai, China; 2 Zhongdai Street Community Health Service Center, Pinghu, China; 3 Department of Cardiology, The First People’s Hospital of Pinghu, Pinghu, China; 4 Department of Emergency Medicine, Sir Run Run Shaw Hospital, Zhejiang University School of Medicine, Hangzhou, China; 5 Department of General Practice, Peking University First Hospital, Beijing, China; 6 Department of General Practice, Peking University Shenzhen Hospital, Shenzhen, China; 7 Department of General Practice, The Second Affiliated Hospital, Zhejiang University School of Medicine, Hangzhou, China

**Keywords:** medication burden, multimorbidity, physician behavior, primary care, qualitative research

## Abstract

**Aims:**

Medication burden represents the effort and stress associated with managing medications, encompassing the physical and psychological impact that patients bear during disease treatment, yet little research has focused on medication burden among patients with multimorbidity in rural areas. This study aims to explore patient experiences of medication burden among those patients with multimorbidity in rural China, and examine how physicians’ behaviors affect this burden.

**Materials and methods:**

This qualitative descriptive study employed in-depth, face-to-face interviews to explore patients’ experiences. Participants were adults with multimorbidity who regularly sought medication-related care at four township health centers in rural China. Data were analyzed using the constant comparative method to identify recurring patterns and generate thematic insights.

**Results:**

Of the 20 participants, 11 (55%) were women, and the mean age was 65.5 years (range: 31–85 years). Participants described various forms of medication burden, including difficulties with daily medication routines, complex regimens, adverse drug reactions, and limited access to medications in primary care settings. Social burdens, such as stigma and restrictions on social activities, were also identified. Participants reported that physicians’ behaviors-both negative (e.g., poor communication, lack of medication review) and positive (e.g., clear medication guidance, follow-up care)-significantly influenced their medication burden.

**Conclusion:**

Patients with multimorbidity in rural China face substantial medication burden, which can be influenced by physicians’ behaviors. Efforts to enhance physicians’ prescribing competencies, improve multimorbidity management, shared decision-making, and follow-up care are essential to alleviate this burden.

## Introduction

Multimorbidity, defined as the co-occurrence of two or more chronic conditions in an individual, is increasingly prevalent and poses substantial challenges to healthcare systems worldwide (1). Patients with multimorbidity often experience multiple medication use, higher rates of hospitalization, greater healthcare utilization, and poorer health outcomes (2). These challenges are particularly pronounced in primary care settings in rural areas, where the demand for integrated, continuous, and patient-centered care is high. Multimorbidity contributes to complex treatment regimens and rising healthcare costs, placing significant demands on both patients and providers (3).

Among the multiple challenges faced by patients with multimorbidity, treatment burden has emerged as a key area of concern in health services research. Treatment burden encompasses the workload of healthcare and its impact on patients’ functioning and well-being (4, 5). This includes time and financial costs, emotional and cognitive strain, and disruptions to daily life. Treatment burden is often intensified by the need to adhere to diverse and sometimes conflicting treatment plans, including medications, lifestyle modifications, and frequent healthcare appointments (6, 7). A key contributor to this burden is polypharmacy, commonly defined as the concurrent use of five or more medications, which is highly prevalent among patients with multimorbidity (8). The risks associated with polypharmacy are further compounded by overprescribing and the absence of deprescribing, leaving patients to manage complex regimens without adequate reconciliation (9). Treatment burden can undermine adherence, negatively affect outcomes, and perpetuate a cycle of poor health (10).

A core component of treatment burden is medication burden. The burden of drug therapy refers to the effort and physical and psychological impact that patients need to bear during the treatment of diseases (11–13). This includes complexity of regimens, risk of adverse drug reactions (ADRs), and concerns about drug–drug interactions. For patients with multimorbidity, particularly those in rural settings, medication burden can be especially challenging due to fragmented care, limited access to healthcare resources, and inadequate support systems (10, 14). In such contexts, the capacity of local healthcare systems to provide effective medication management is often constrained, further exacerbating the challenges faced by patients (15).

Existing studies have highlighted the significant impact of medication burden on treatment adherence and health outcomes. Research has shown that high medication burden is associated with decreased adherence to prescribed regimens, which leads to poorer health outcomes (16). Studies have identified various factors contributing to medication burden, including complexity of regimens, side effects, and the financial strain associated with obtaining medications (17). There is also growing recognition that medication burden not only affects physical health but also has psychological and social impacts, such as increased stress and social isolation (18). Despite of these findings, there remains a gap in understanding medication burden faced by patients with multimorbidity in rural areas of China. The challenges of managing medication burden in rural areas are exacerbated by limited resources and service capacity, further complicating the multimorbidity care (10, 15).

Previous studies have shown that physicians’ behaviors play a significant role in prescribing practices. For instance, effective communication has been found crucial in prescribing practices as it helps ensure that patients understand their treatment plan, potential side effects, and the importance of adherence (19). Inappropriate prescribing practices, such as overprescribing or failing to consider drug interactions, are more common when physicians do not take a comprehensive approach to managing patients with multimorbidity (20). However, few studies have specifically explored the relationship between physicians’ behaviors and patients’ medication burden, particularly in the context of multimorbidity in rural areas. These behaviors may be prevalent in primary care settings, where physicians often focus on treating individual conditions without considering the cumulative impact of treatment on patients’ burden.

In rural China, primary healthcare is delivered through township health centers, where general practitioners serve as the first point of contact for patients with multimorbidity. These centers operate under a national essential medicines policy that limits the availability of certain medications, often requiring patients to obtain prescriptions from higher-level hospitals. Referrals to specialists typically occur through a tiered system, which can result in fragmented care and limited coordination across providers (21). Understanding this healthcare context is critical for interpreting patients’ experiences of medication burden. This study aims to explore the experiences of medication burden among patients with multimorbidity in rural China and to analyze the impact of physicians’ behaviors on this burden. It seeks to provide insights for policymakers and primary care providers to optimize community-based medication management services and inform strategies that reduce treatment burden for patients with multimorbidity in rural areas.

## Methods

### Study design

This study employed a qualitative descriptive methodology, appropriate for exploring patients’ experiences of medication burden. Semi-structured, in-depth interviews were conducted with all participants, each of whom provided written informed consent prior to participation. To ensure methodological rigor and transparency, the study was conducted in accordance with the Consolidated Criteria for Reporting Qualitative Research (COREQ) guidelines (22). Ethical approval was obtained from the Ethics Committee of the Second Affiliated Hospital of Zhejiang University School of Medicine. All procedures were conducted in accordance with the Declaration of Helsinki, upholding the principles of respect, integrity, and confidentiality for all participants (23).

### Setting and study population

The study was conducted within China’s three-tier rural healthcare system, where township health centers serve as the primary care hub. Unlike Western general practice, Chinese rural physicians working in these centers act as both clinicians and pharmacists because integrated community pharmacy services are absent. These facilities operate under the National Essential Medicines List policy with zero-markup pricing, which frequently results in medication shortages, which forces patients to travel to county hospitals for prescriptions. While formal referral pathways exist between tiers, they lack gatekeeping enforcement. Patients often bypass rural doctors to self-refer to specialists, which contributes to fragmented care and duplicate prescribing.

A purposive sampling approach was employed to select participants for this study (24). Eligible individuals were aged 18 years or older, had been diagnosed with two or more chronic conditions, were concurrently using two or more medications, and had received care for at least 6 months at one of four township health centers located in rural areas in Zhejiang Province. Exclusion criteria included the presence of terminal illnesses, malignancies, dementia, or unwillingness to participate in or be recorded during the interview. Eligible patients were identified via electronic health records, and sampling diversity was ensured by stratifying by age, number of medications, and education level to cover varied experiences.

Recruitment was conducted in collaboration with general practitioners at the township health centers. No relationship established between interviewers and participants prior to the study. The study’s purpose and procedures were explained to potential participants during preliminary interactions, either face-to-face or via WeChat, a widely used social media application in China. Interview appointments were arranged at least 2 days in advance to accommodate participants’ schedules.

Of the initially contacted individuals, three declined participation due to scheduling conflicts. All other participants voluntarily consented to participate and were given a small token of appreciation (approximately USD $7). No follow-up or repeat interviews were conducted.

### Interview guide

The semi-structured interview guide was developed through an extensive review of the relevant literature and pilot interviews conducted with two patients with multimorbidity (25–27). Feedback from experts in primary healthcare was incorporated into the guide. Following iterative discussions among the research team members, the final version of the interview guide was established (see Supplementary material-interview guide). The guide included several main questions designed to prompt discussion on key topics, supplemented by probing questions to elicit more detailed responses and clarify participants’ perspectives. The interview questions focused on two principal domains: (1) participants’ experiences of medication burden and (2) the influence of physicians’ behaviors on participants’ experiences of medication burden.

Participants were also encouraged to share any additional thoughts or comments they considered relevant. Iterative reflection and minor adjustments to the guide were made throughout the interview process to enhance its effectiveness and relevance.

### Data collection

Between November 1, 2023, and January 10, 2024, two interviewers-a female and a male -both experienced general practitioners trained in qualitative research methods, conducted the interviews. Participants were contacted by their family physicians via phone, WeChat, or in-person to schedule interviews at mutually convenient times. To facilitate familiarity with the discussion topics, the interview guide was sent to participants 2 days prior to their scheduled interview. Interviews were conducted in quiet, private settings to promote open and candid discussions.

At the beginning of each interview, the study’s objectives, procedures, and potential impacts were explained in detail. Participants were encouraged to ask questions, and written informed consent was obtained before proceeding. Basic demographic and clinical information was collected to contextualize participants’ responses, with all necessary precautions taken to protect confidentiality and minimize the risk of inadvertent disclosure.

The interviewers adopted a flexible, participant-centered approach, adjusting the sequence and phrasing of questions based on the flow of each conversation. Open-ended and probing questions, such as “Why did you experience this?” and “Can you provide an example?”, were used to elicit rich, detailed narratives. Interviewers maintained a respectful, nonjudgmental stance throughout and refrained from providing encouragement or interjections to minimize interviewer bias.

All interviews were audio-recorded using iFlytek SR501 devices and lasted between 30 and 45 min. Key observations, including non-verbal cues, were documented in field notes to complement the recordings. Each participant was assigned a unique numerical code, and all personal identifiers were removed to ensure anonymity and maintain the integrity of the data collection process.

### Reflexivity

Both interviewers were general practitioners with clinical experience in managing patients with multimorbidity in rural settings. Their professional background may have facilitated rapport with participants by fostering trust and mutual understanding of healthcare contexts, enabling richer accounts of medication-related experiences. However, this shared professional identity may also have influenced participants to present their medication practices in a more favorable light, contributing to potential social desirability bias. To mitigate this, interviewers emphasized the study’s non-judgmental purpose, assured participants of confidentiality, and encouraged candid discussion of challenges in medication management.

### Data processing and analysis

Interview data were transcribed verbatim within 24 h of each interview and subsequently checked for accuracy. Field notes documenting non-verbal cues and key observations were integrated into the data analysis process to enhance contextual understanding, as they provided additional context for interpreting participants’ verbal responses. Two researchers, both extensively trained in qualitative data analysis, independently conducted line-by-line coding of the transcripts. During this process, initial open codes were generated, discussed, and refined, leading to the development of a preliminary codebook (28).

The coding framework was refined iteratively as new categories and themes emerged from the data. Inter-coder consistency was established through regular consensus meetings, where discrepancies were resolved via discussion until full agreement was reached, and a third researcher was consulted when consensus could not be immediately achieved. A constant comparative method was employed to continuously develop and revise the thematic framework until no new subcategories were identified (29). Data saturation was achieved after 16 interviews, indicating that no substantially new information regarding medication burden experiences was emerging. Four additional interviews were conducted to ensure comprehensive thematic coverage and confirm saturation. Although participants did not review the full interview transcripts, five participants were invited to review the study’s findings (e.g., thematic summaries and representative quotes) to validate the interpretations and ensure they accurately reflected participants’ experiences. Data analysis was facilitated using MAXQDA 2022 software (VERBI Software, Berlin).

To preserve the authenticity of the data, all interviews were transcribed in Mandarin. The transcripts underwent an iterative process of translation and verification. First, they were translated into English by a bilingual researcher. This initial translation was then cross-checked through back-translation by an independent linguist. Any inconsistencies identified between the back-translated Chinese and the original transcripts were thoroughly discussed and reconciled by the research team to guarantee the precision and contextual integrity of the final English version.

## Results

A total of 20 participants were enrolled in the study. All participants were of Han ethnicity, and 11 (55%) were female. The mean age of participants was 65.5 years, with ages ranging from 31 to 85 years. Details of patient characteristics and thematic summary of key findings are documented in the Supplementary Table S1 and Supplementary Figure S1.

### Experience of medication burden among patients with multimorbidity

#### Daily medication burden

Participants described challenges in coordinating their medication schedules with daily activities, particularly when doses conflicted with meal times or work routines. The challenges are related to managing multiple medications concurrently. These burdens included coordinating multiple dosing schedules, distinguishing between tablets with similar appearances, and remembering varied instructions. Such challenges reflect the cumulative complexity of polypharmacy rather than the characteristics of any single drug. Many older participants reported issues connected to memory, including forgetting to take medications, taking medications multiple times, or accidentally ingesting the wrong ones.

“Taking so many medications means I can’t work in the fields anymore. If I do go out, it’s hard to keep track of time and get back home to take my meds on schedule. Sometimes I forget my watch, and then I lose track of time. When that happens, I either miss a dose or, if I take my diabetes meds and go to work, I risk having low blood sugar” [Participant 12].

Negative emotions associated with medication use were frequently mentioned, such as frustration over complex regimens, hopelessness about having to stay on medication indefinitely, and irritability when medication routines disrupted daily life.

“I take more than ten pills every day. Watching others stay healthy while I’m stuck relying on medications makes me feel like a failure. I feel like I’m just a walking medicine jar. Sometimes I take more pills than I eat food. It’s really depressing” [Participant 3].

#### Medication regimen complexity

Participants reported that the complexity of their medication regimens created problems, particularly given the need to manage multiple chronic conditions simultaneously. Specific difficulties included distinguishing between medications with similar appearances, remembering the correct dosages, and managing different administration methods (e.g., oral, injectable, inhaled, or brewed formulations). Participants using insulin or inhalers highlighted that these therapies required distinct technical skills and environmental controls (e.g., refrigeration, hand-lung coordination) that were not necessary for their oral medications. Some participants also described physical barriers, such as difficulty swallowing large tablets.

“I have to take so many medications every day that I often forget some during a single dose. Sometimes I can’t even remember which ones I’m supposed to take. Since I can’t read, I once mistook a bottle of mouthwash for cough syrup and drank it” [Participant 5].

“I need to take more than ten medications a day, and they all look the same. I’m old, my eyesight’s bad, and I have difficulties in reading. It’s really hard to tell them apart, so I often end up taking the wrong one” [Participant 9].

#### Adverse drug reactions

Participants described experiencing a range of burdens associated with ADRs, from mild and tolerable symptoms to severe, life-threatening complications. Some reported that ADRs caused significant physical discomfort or led to changes in their medication behaviors. For instance, participants noted experiencing physiological discomfort, abnormal laboratory results, or worsening conditions due to side effects, which often necessitated additional medications to manage these reactions. A few participants reported altering or discontinuing their prescribed regimens due to fear of ADRs or prior negative experiences.

“My joints hurt so badly that I have to take painkillers all the time. But the painkillers upset my stomach, giving me frequent stomachaches and acid reflux, so now I have to take medication for my stomach issues too”[Participant 13].

Most participants described ADRs in terms of physical discomfort, such as gastrointestinal symptoms or abnormal laboratory results. However, one pregnant participant highlighted an additional concern.

“I’m pregnant now, and I’m really worried about how the medications might affect the baby. Before, I was very resistant to taking any medications. When my blood sugar was high, I took metformin for two or three days and then stopped because I was scared it would harm the baby. Later, I switched to insulin. During early pregnancy, my liver function was not good, and the doctor prescribed medications, but I stopped that after two days out of fear for the baby” [Participant 10].

#### Healthcare-related burden

Participants described several healthcare related burden, including the limited availability of medications in primary care settings, restrictive insurance policies, and communication barriers with healthcare providers. These structural constraints were frequently exacerbated by the rural healthcare infrastructure, where fragmented care delivery and resource limitations compounded access difficulties. Some participants reported having to visit multiple specialists to obtain their prescribed medications, while others complained with the restrictions on the quantity they could obtain at each visit.

“The inhaler I use for bronchitis isn’t available at the township health center, so I have to go to the hospital in the city to obtain the prescription. But in the hospital, you have to go to the respiratory department first, which means waiting in long lines and wasting half a day” [Participant 11].

“I have this one med for my heart, and insurance won’t let me pick up more than 15 days’ worth. But I can’t skip it. If I miss even a day, I feel unwell. So every two weeks, I have to make the trip to the hospital again. It’s exhausting, especially when my son is busy and can’t take me” [Participant 8].

#### Social burden related to medications

Some participants reported experiencing stigma or reduced social interactions as a result of their medication use within workplace and community contexts. A few described being avoided or discriminated against by neighbors or coworkers, particularly in agricultural work settings where health conditions were sometimes misinterpreted as contagious or disabling, leading them to conceal their medical conditions and medication use to avoid negative perceptions. Others noted that the timing or storage requirements of certain medications restricted their ability to travel or participate in social activities. Social events occasionally caused participants to miss doses or take medications incorrectly, reflecting broader societal expectations regarding productivity and social participation in rural communities.

“When I first started taking medications, my coworkers avoided me because they thought I was taking psychiatric drugs. They would tell others not to talk to me, and some people would just stare, point fingers, and refuse to speak to me” [Participant 3].

“I take so many medications that it really affects my daily life, especially when it comes to meals with family. For example, when I visit relatives, their meal times aren’t fixed, but I have to inject insulin 30 minutes before eating. I can’t expect them to change their schedule just for me. And traveling long distances is hard because I need to refrigerate my insulin. Now, I don’t even visit my sister or my daughter anymore” [Participant 9].

### Physician behaviors influencing the medication burden

#### Lack of medication assessment

As patients’ conditions frequently fluctuate, regular assessments and adjustments to treatment regimens are essential. However, a minor of participants reported that physicians failed to conduct adequate assessments or tailor prescriptions to patients’ changing health status. Several participants noted that doctors simply replicated previous prescriptions without reassessing their conditions, leading to poor disease control and unnecessary medication use.

“When I go to the township health center to refill my prescription of antihypertensive drugs, the doctor sometimes but not every time, checks my blood pressure and gives me the same medications as before. But my blood pressure has been poorly controlled for a long time. The doctor seems helpless and hasn’t made any adjustments to my medications” [Participant 16].

“Some doctors don’t even ask how well my condition is being managed. They just prescribe the same medications as last time without checking anything. It feels like they don’t even know or care whether my condition has gotten better or worse” [Participant 12].

Participants also observed that the complexity and overlap of their diseases and medications often necessitated comprehensive evaluations and integrated treatments, which many physicians were unable to provide. Instead, patients were frequently referred to different specialists for separate conditions. Several participants noted that physicians tended to focus narrowly on specific symptoms without considering the broader clinical context.

“You go to one specialist for stroke, a completely different one for hypertension. Nobody’s looking at the whole picture. But a lot of these conditions actually happen together. Once one issue is dealt with in one department, I have to go to another department for the other problem” [Participant 1].

#### Poor communication

Participants emphasized the lack of shared decision-making and insufficient communication regarding their medications. Many patients reported having numerous questions about their conditions and treatments, making effective doctor–patient communication particularly critical. However, older participants frequently cited language barriers, noting that some physicians were unable to communicate in the local dialect, thereby hindering discussions about their health and treatment plans.

“I can’t speak Mandarin very well, and some doctors don’t speak my local dialect. So they don’t understand me, and I don’t understand them. We just can’t communicate at all” [Participant 17].

Some participants also reported that physicians rarely involved them in decisions about their medications. Physicians often failed to ask about patient preferences or explain the potential side effects of prescribed drugs. Participants expressed that, had they been informed of possible adverse effects, they might have declined certain treatments. In rural areas, many patients perceived a “good” physician as one who fulfilled all their medication requests, regardless of medical appropriateness. Participants noted that some doctors prescribed medications solely based on patient demands.

“Community doctors are usually very nice. If I ask for a specific medication, they’ll prescribe it. Whatever I say, they’ll give it to me. If I want to switch to a different medication, they’ll do it without asking any questions” [Participant 13].

Participants also stressed the critical role of medication education. Patients with multimorbidity often follow complex treatment regimens, and proper guidance is essential to ensure the safe and effective use of medications. However, some participants reported that physicians rarely provided detailed instructions on medication use or strategies for managing potential side effects. For example, one patient on long-term antidiabetic therapy mentioned never being informed about the risk of hypoglycemia, leading to missed opportunities for appropriate care during episodes of low blood sugar. Others described how physicians often assumed that patients would know how to use their medications correctly without offering any explicit instructions. As a result, some participants had to return to the clinic, sometimes multiple times, to seek clarification.

“Some doctors prescribe medications but don’t explain anything. When I get home, I have no idea how to take them. So then I have to go back to the hospital, sometimes multiple times, just to ask the doctor how to use them. Sometimes the doctor gets impatient, and that just makes things even more troublesome” [Participant 10].

While participants primarily focused on physicians’ behaviors, some noted that other healthcare providers, such as nurses, occasionally provided medication guidance or injection training. However, the involvement of pharmacists or structured interprofessional collaboration was rarely mentioned, reflecting the limited integration of multidisciplinary teams in rural primary care settings.

#### Inappropriate prescribing

Some participants reported that certain physicians prescribed unnecessary medications. One participant noted that doctors issued overly extensive prescriptions, causing confusion and increasing the risk of medication errors. She also mentioned being prescribed traditional Chinese medicines she did not need.

“When I go to refill my prescriptions, some doctors don’t ask about my condition and just rush through the process. Sometimes, after giving me the medications I actually need, they prescribe extra ones I don’t need, like some traditional Chinese medicines. Having so many medications confuses me, and it’s easy to take them incorrectly” [Participant 10].

Participants also highlighted inconsistencies in the professional competence of primary care physicians. A few described experiences where doctors made mistakes in prescribing medications or determining dosages. Some participants felt that certain physicians lacked adequate medical knowledge, leading to incorrect prescriptions.

“I once visited the township health center for a cold. The doctor prescribed a lot of medications, including levofloxacin. He told me to take one tablet three times a day. But since I have a habit of reading medication instructions, I noticed it was supposed to be taken only once a day” [Participant 8].

#### Medication education

Some participants reported receiving helpful medication education from their physicians following the prescription of medications. They appreciated the physicians’ patience in guiding them on the proper use of specific drugs, particularly those requiring special techniques, such as insulin and inhalers. Doctors explained administration methods, dosage, frequency, and potential side effects in detail, enabling patients to use their medications safely and effectively to achieve optimal therapeutic outcomes.

“The doctor taught me how to inject insulin and explained that it has to be given on time. They also told me that while using insulin, I should keep my meals consistent, no overeating or skipping meals, to avoid side effects like hypoglycemia” [Participant 19].

“I use an inhaler for my bronchitis, and the doctor patiently showed me how to use it. They taught me how to inhale and exhale properly and reminded me to rinse my mouth afterward. They explained that not rinsing could cause discomfort or even a fungal infection. I’ve followed their advice and never had any problems like that” [Participant 15].

#### Follow-up

Regular follow-up was identified as a key component of physicians’ responsibilities. Some participants noted that doctors used phone calls, WeChat messages, and even home visits to maintain contact with patients. Remote follow-ups, conducted via text, voice, or video calls were considered particularly convenient. These methods enabled physicians to quickly assess medication adherence, respond to questions, and address any issues arising during recovery. For patients requiring additional support, home visits allowed doctors to evaluate recovery status in person and provide individualized medical advice. Participants also noted that remote follow-ups reduced the social stigma sometimes associated with frequent hospital visits.

“The doctor gave me their phone number and WeChat. Every month, they check in on how I’m doing. If I have any medication issues at home, I can contact them directly, and they explain things clearly. Otherwise, every time I go to the hospital, my neighbors ask why I’m going so often” [Participant 6].

#### Individualized medication management

Medication plans were sometimes adjusted based on patients’ individual needs to reduce treatment burden during consultations. Participants reported that physicians inquired about their current medication use, assessed adherence, and explored barriers to following instructions. One participant shared that he had deliberately skipped medications due to financial constraints, prompting his physician to revise the plan and prescribe a more affordable alternative. In addition, many physicians provided long-term prescriptions for patients with mobility limitations, reducing the need for frequent hospital visits.

“My doctor is very considerate. They always ask if I’ve been taking my medications properly. Since I live in a rural area, traveling to the hospital is inconvenient. When my condition is stable, they prescribe enough medications to last longer so I don’t have to make frequent trips” [Participant 20].

“The medication for my chronic bronchitis is very expensive, so I didn’t follow the doctor’s instructions to use it daily. Every time I saw the doctor, they asked why a one-month prescription lasted two months. After realizing I wasn’t adhering to the treatment, they switched me to a cheaper alternative” [Participant 15].

## Discussion

This qualitative study investigated the medication burden experienced by patients with multimorbidity in rural areas in China, with a particular focus on how physicians’ behaviors influence this burden. The findings demonstrate that medication burden not only shapes patients’ perceptions and behaviors regarding their treatment regimens but also exerts a substantial impact on their overall health and well-being. Moreover, physicians’ behaviors were found to have diverse effects, either mitigating or exacerbating the medication burden among these patients.

Our study found that patients with multimorbidity in rural areas face challenges in obtaining and consistently using multiple prescribed medications, and their experiences of medication burden are marked by both variability and complexity. Consistent with previous research, fragmented healthcare resources, limited drug availability, and high medication costs present substantial barriers to medication access and adherence in these settings (30). For example, A study conducted in a Chinese community reported that more than 75% of the elderly with multiple diseases need to take more than five kinds of drugs (31). Moreover, the complexity of medication regimens characterized by the large number of medications and inconsistent administration instructions, often leads to confusion, missed doses, or other medication errors (32). Our study reveals how medication burden manifests in daily life through physical, cognitive, emotional, and financial dimensions, offering a deeper understanding of the mechanisms behind the attitudes and preferences identified in quantitative research.

A further distinction merits consideration. Some of the medication burdens reported by participants arise from the inherent complexity of specific drugs, whereas others reflect the cumulative challenges of managing multiple medications concurrently. Although these two dimensions are conceptually distinct, in patients with multimorbidity they often intersect and amplify one another. For example, the need to coordinate insulin timing with other oral medications while managing competing demands from multiple chronic conditions illustrates how drug specific burdens become magnified within the context of polypharmacy (7). The critical challenge in multimorbidity lies in the interaction between these specific demands and the overarching complexity of polypharmacy. Recognizing this distinction is important for designing targeted interventions. Some strategies may focus on simplifying regimens through deprescribing or medication synchronization, while others may address drug specific barriers through patient education or device optimization (9, 33).

Our findings confirm and extend established medication burden frameworks from previous studies. While existing frameworks focus on patient-level factors (medication complexity, financial burden, side effects), our study reveals additional negative factors and systemic constraints specific to rural healthcare contexts. For example, lack of medication assessment emerges as a critical factor not fully captured in existing frameworks. This is compounded by clinical decision fatigue, which significantly impacts healthcare providers’ ability to conduct thorough assessments in resource-constrained environments (34). Poor physician-patient communication represents another significant factor beyond traditional complexity measures. Communication deficits include inadequate medication explanations, limited patient question time, and complex medical terminology. This aligns with evidence showing physicians adapt communication practices suboptimally under time and resource pressures (35). The absence of electronic intelligent tools creates significant barriers to medication management. Unlike urban systems with computerized order entry and clinical decision support, rural townships relied on paper-based processes, increasing medication errors and duplicative prescribing. The lack of integrated information systems prevents effective medication reconciliation across providers, creating environments where even competent physicians struggle to provide optimal care.

In our study, the association between medication burden and quality of life among patients with multimorbidity was repeatedly emphasized. An European study found that ADRs pose a significant concern for patients with multimorbidity, especially older adults, as frequent drug–drug and drug–disease interactions can substantially impair their quality of life (36). A qualitative study identified the complexity of medication regimens, time-consuming medication routines, and financial burdens as key concerns expressed by patients (37). In rural areas, medication burden is particularly pronounced due to the lack of basic healthcare infrastructure and unstable medication supply chains, which further hinder patients’ access to essential medications (38).

This study specifically examined whether physicians’ behaviors influence patients’ experiences of medication burden. Participants frequently expressed dissatisfaction with physicians who failed to perform adequate clinical evaluations or to adjust prescriptions appropriately. Such practices were perceived to contribute to poorly controlled health conditions, unnecessary or excessive medication use, increased financial burden, and a higher risk of ADRs. Previous research has shown that in primary care settings, medication errors are frequently associated with human factors such as performance defects, non-compliance with protocols, and inadequate checking procedures, with patients on multiple medications being particularly vulnerable (39). One key contributing factor is clinical inertia, wherein physicians continue previous prescriptions without sufficient reassessment or adjustment during follow-up consultations (40). While clinical inertia is often attributed to physician-related factors such as their limited knowledge of medicines optimization, patient-related factors may also play a role. For instance, our prior study found that some primary care physicians yielded to patient demands or perceived pressure to prescribe unnecessary medications, which reinforced clinical inertia and compromised appropriate medication management (41).

Participants in our study reported that some physicians prescribed inappropriate medications, which contributed to increased medication burden. In addition, poor communication from physicians (e.g., the use of unfamiliar medical terminology or local language barriers) was frequently cited as a source of confusion, leading to improper medication use among rural patients. Notably, many participants equated a “good doctor” with one who fulfills all patient requests, even when such requests were medically inappropriate, reflecting a misunderstanding of professional roles and therapeutic decision-making. Prior research has demonstrated that shared decision-making is especially important for patients with multimorbidity, as it enhances trust in physicians, increases satisfaction with care, and promotes adherence to often complex treatment plans (42, 43). In contrast, a lack of shared decision-making has been associated with greater medication burden and decreased patient satisfaction and compliance (44). One commonly reported obstacle to implementing shared decision-making is limited consultation time, which underscores the importance of improving continuity of care and patient-physician communication to support informed and appropriate treatment decisions.

With the advancement of internet technology, patient follow-up has become increasingly convenient and diversified. Previous studies have shown that regular follow-up is essential in chronic disease management, as it significantly reduces the risk of adverse health outcomes (45, 46). In our study, physicians maintained contact with patients through a combination of home visits, phone calls, and the messaging application WeChat. These approaches enabled timely communication, allowing physicians to address patient concerns promptly. Moreover, they helped alleviate both the financial and logistical burdens associated with frequent hospital visits, while also reducing the social stigma that some patients may experience when seeking care in formal medical settings.

Individualized care is essential for reducing medication burden among patients with multimorbidity. In our study, participants reported that empathetic physicians who adjusted medication regimens in accordance with patients’ financial and logistical constraints were more effective in alleviating their overall burden. The meta-analysis revealed a slight but statistically significant 8% reduction in hospital readmissions following medication review and deprescribing (47). Individualized care has been shown to be critical in reducing treatment burden among patients with multimorbidity, where complex regimens and competing priorities often lead to non-adherence and suboptimal outcomes (48). These findings underscore the necessity of tailoring treatment plans to individual needs and preferences, and of adopting a holistic, patient-centered approach to care (9). To support this goal, it is crucial to develop comprehensive training programs for primary care physicians that enhance their ability to improve medication management and reducing patient burden. Beyond the role of physicians, interprofessional collaboration has been shown to reduce treatment burden by providing medication education, adherence support, and medication reconciliation (49). However, in our study, participants rarely reported such interactions, highlighting a gap in team-based care within rural health systems. Strengthening interprofessional approaches may offer additional opportunities to alleviate medication burden in resource-limited settings.

Our findings have several implications for targeted interventions in rural healthcare settings. Training programs for primary care physicians that enhance their ability to apply clinical guidelines effectively in rural areas, thereby improving medication management and reducing patient burden, should be prioritized. These programs should address not only clinical knowledge but also strategies for managing clinical decision fatigue and developing systematic approaches to medication review in complex cases. Healthcare systems should consider implementing structured medication review protocols, establishing telemedicine platforms for specialist consultation, and developing integrated care pathways that facilitate coordination among multiple providers. Investment in health information systems that support medication reconciliation and clinical decision support could help mitigate the impact of systemic constraints on prescribing behaviors. Additionally, addressing workload distribution and providing adequate time for patient consultations are essential policy considerations for reducing medication burden in rural settings.

### Limitations

This study has several limitations that should be considered when interpreting the findings. First, participants were recruited from patients attending four health centers in China. This geographic concentration may limit the generalizability of the results to other rural regions or broader healthcare systems. Nevertheless, to enhance sample diversity, we employed a maximum variation sampling strategy, selecting participants from 15 villages served by the four township health centers. Second, the qualitative nature of the study limits its ability to quantify the extent of medication burden or establish causal relationships between physician behavior and patient outcomes. However, data saturation achieved after 16 interviews and rigorous constant comparative analysis ensure that the identified themes represent shared experiential patterns rather than isolated individual narratives. Further quantitative studies are needed to validate and extend these findings. Third, the reliance on self-reported data may introduce recall bias or social desirability bias, particularly in participants’ descriptions of physician interactions and medication practices. Fourth, the cross-sectional design captures participants’ experiences at a single point in time, without accounting for potential changes in medication burden or physician-patient dynamics over time. Future research using longitudinal or intervention-based designs is warranted to evaluate the effectiveness of these strategies in reducing medication burden in similar resource-limited settings, which can provide more robust evidence for policy-making and practice.

## Conclusion

Patients with multimorbidity in rural China face substantial and complex medication burdens, and physicians’ behaviors play a critical role in either alleviating or exacerbating this burden. Enhancing physicians’ competencies in rational prescribing and multimorbidity management, promoting shared decision-making, strengthening follow-up care and interprofessional collaboration are essential strategies for reducing medication burden and improving patient outcomes in resource-limited contexts.

## Data Availability

The raw data supporting the conclusions of this article will be made available by the authors, without undue reservation.
