# Supplementary material for: Experiences of medication burden among patients with multimorbidity in rural China: a qualitative study
Source: Front Med (Lausanne). 2026 Apr 21;13:1772444. doi: 10.3389/fmed.2026.1772444 (PMC13138948; doi:10.3389/fmed.2026.1772444)
Supplement: Supplementary file 1 [file Table_1.DOCX]

### **Supplementary Material**

**Interview guide**

**I. Introduction**

Welcome to the interview. I am a general practitioner who has long been concerned with medication issues for patients with multiple chronic diseases. I know that over the years, you have taken many medications, which have played a crucial role in controlling your condition; however, taking these medications has also brought you some troubles and burdens. Therefore, in this interview, I will ask you some questions related to this aspect. Please share your experiences with me based on your real situation and share your thoughts. Your answers are not right or wrong, and these questions are not specifically directed at you. There is no need to answer in a particular way. If you find one of my questions inappropriate, you can refuse to answer it, and you have the right to terminate the interview at any time.

Do you have any other questions to communicate before our interview officially begins?

I will start recording now and write down some of the key information you mentioned on paper.

**II. Interview framework**

*1. Disease status*

A. Are you in good health now? Can you talk to me about your current chronic diseases? Which disease did you get first? What diseases did you get later?

B. How has your condition or indicators been controlled recently?

*2. Multimorbidity management*

A. I would like to know how you manage your illness on a daily basis.

1. What do you need to do every day to better control your condition?
2. Can you walk me through a typical day of managing your medications?
3. What experiences have you had during your medication use process?
4. What else have you done to alleviate your condition?
5. You just mentioned that there are many difficulties in the process of taking medicine. How have these things affected you?
6. (You mentioned that the first disease you got was...), what impact did the drugs used for that disease have on your work and life?
7. What effect does it have on your mood?
8. How do you overcome these negative effects?
9. Are you financially able to treat the disease? Does it have a big impact on your family's financial situation?
10. Now I will ask you about the second disease and medication. (What effect does this disease have on the previous diseases and medications you have taken? Repeat a-e)
11. (After asking all the diseases) Do you have any of the above problems in your examination and treatment? What difficulties do you have in using drugs at the same time compared with a single drug? Why do you think this effect will occur?

*3. Physician behaviors*

A. Thinking about the doctors you see for your medications, how would you describe your communication with them?

a. Can you give me an example of a time when communication with a doctor was particularly helpful or unhelpful for managing your medications?

b. When a new medicine is prescribed, how does the doctor typically explain how to use it and its potential side effects?

c. When you go for a follow-up visit, what does the doctor typically do to check if your medications are still working well for you?

d. Have you ever felt that your condition (e.g., blood pressure, pain) was not well-controlled, but the doctor simply renewed your old prescription without making changes?

e. In your experience, do you feel that doctors consider all of your health conditions together when prescribing, or do they focus on them one by one?

f. Can you tell me about a time when you felt this approach was a problem or particularly helpful?

B. Beyond writing a prescription, what kind of support do doctors provide to help you manage your medications?

a. Have any doctors provided follow-up support, for example, through phone calls, WeChat, or home visits? How did that impact you?

b. Have doctors ever adjusted your medication plan to make it easier for you, for instance, by considering cost or making it simpler to take?

c. Overall, what actions by doctors make it easier for you to manage your multiple medications? What actions make it more difficult?

**III. Conclusion**

I have basically finished my questions. Do you have anything else to add about your medical condition and other related experiences of seeking medical treatment and taking medicine?

Thank you for your cooperation in this interview. I feel very happy to talk with you. Your experience and views have given us new inspiration to further understand the management of chronic disease co-morbidity. I hope we can keep in touch in the future!

**Table S1** Participant characteristics

| **Number** | **Gender** | **Age** | **Educational background** | **Number of medications** |
| --- | --- | --- | --- | --- |
| 1 | F | 75-79 | Primary school | 7-9 |
| 2 | M | 65-69 | Primary school | 7-9 |
| 3 | F | 55-69 | Junior high school | 4-6 |
| 4 | F | 50-54 | Junior high school | 4-6 |
| 5 | F | 65-69 | illiterate | 10-12 |
| 6 | F | 30-34 | Junior high school | 4-6 |
| 7 | F | 50-54 | Junior high school | 4-6 |
| 8 | M | 70-74 | High school | 4-6 |
| 9 | M | 85-89 | Primary school | 10-12 |
| 10 | F | 30-34 | college | 4-6 |
| 11 | M | 75-79 | illiterate | 7-9 |
| 12 | F | 75-79 | illiterate | 4-6 |
| 13 | F | 75-79 | Primary school | 7-9 |
| 14 | F | 65-69 | Primary school | 7-9 |
| 15 | M | 65-69 | Junior high school | 4-6 |
| 16 | M | 80-84 | illiterate | 7-9 |
| 17 | M | 70-74 | Primary school | 4-6 |
| 18 | M | 70-74 | Primary school | 4-6 |
| 19 | M | 75-79 | Primary school | 7-9 |
| 20 | F | 50-54 | Junior high school | 4-6 |


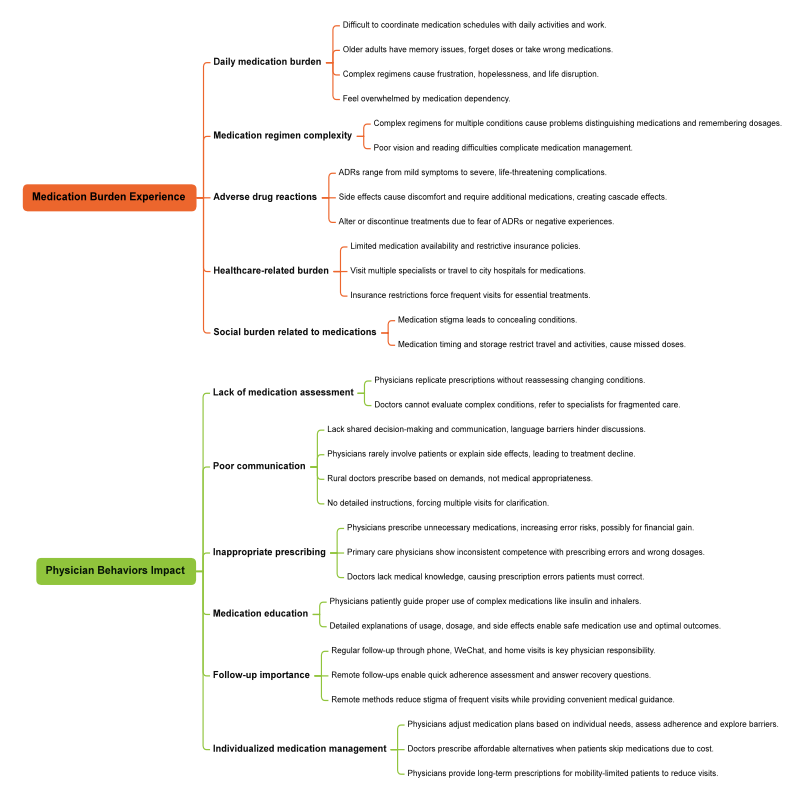


**Figure S1** Thematic summary of key findings
